# Magnesium Coated Bioresorbable Phosphate Glass Fibres: Investigation of the Interface between Fibre and Polyester Matrices

**DOI:** 10.1155/2013/735981

**Published:** 2013-08-27

**Authors:** Xiaoling Liu, David M. Grant, Andrew J. Parsons, Lee T. Harper, Chris D. Rudd, Ifty Ahmed

**Affiliations:** Division of Materials, Mechanics and Structures, Faculty of Engineering, University of Nottingham, Nottingham NG7 2RD, UK

## Abstract

Bioresorbable phosphate glass fibre reinforced polyester composites have been investigated as replacement for some traditional metallic orthopaedic implants, such as bone fracture fixation plates. However, composites tested revealed loss of the interfacial integrity after immersion within aqueous media which resulted in rapid loss of mechanical properties. Physical modification of fibres to change fibre surface morphology has been shown to be an effective method to improve fibre and matrix adhesion in composites. In this study, biodegradable magnesium which would gradually degrade to Mg^2+^ in the human body was deposited via magnetron sputtering onto bioresorbable phosphate glass fibres to obtain roughened fibre surfaces. Fibre surface morphology after coating was observed using scanning electron microscope (SEM). The roughness profile and crystalline texture of the coatings were determined via atomic force microscope (AFM) and X-ray diffraction (XRD) analysis, respectively. The roughness of the coatings was seen to increase from 40 ± 1 nm to 80 ± 1 nm. The mechanical properties (tensile strength and modulus) of fibre with coatings decreased with increased magnesium coating thickness.

## 1. Introduction

Bioresorbable polymers have shown great potential in orthopaedic applications due to their advantages over traditional metals and alloys such as allowing gradual transfer of loads to the healing bone, in order to reduce or eliminate stress shielding effects and avoiding secondary surgery for removal [1]. Stress shielding in bone repair applications occurs as the result of the reduction of stress from the bone via use of an implant. A variety of resorbable polymers have been identified as suitable for clinical use in orthopaedics, such as the polyesters: polylactic acid (PLA), polyglycolic acid (PGA), polycaprolactone (PCL), and their copolymers. However, the mechanical properties of these resorbable polymers are often insufficient especially for load bearing bone repair applications. As such reinforcing resorbable polymers is an attractive approach to overcome these limitations, which can be obtained via fabrication of fibre reinforced composites.

Bioresorbable phosphate glass fibre (PGF) reinforced polymer composites have attracted much interest, and researchers investigating these composites have indicated that the mechanical properties of these materials are easily tailored by adjusting their fibre volume fraction [2–4]. However, studies *in vitro* have shown an initial rapid loss of composite mechanical properties in the very early stages of immersion within aqueous media. This phenomenon is believed to be due to plasticisation of the matrix and degradation of the interface between the fibres and matrix when exposed to an aqueous media [2–5]. 

The interface is crucial for the performance of the composites, and typically, interfacial adhesion can be improved by chemically linking the glass fibre and polymer or via mechanical interlocking between the fibre and the matrix [6]. Methods used successfully to improve interfacial adhesion in glass fibre-reinforced polymers have involved coupling agent treatments [7–9], plasma etching [10, 11], and plasma polymerisation [12–15] of fibres. However, improving the interfacial strength in biodegradable systems has proved rather challenging due to the degrading nature of the materials concerned. The phosphate glass fibre/PLA interface studied by Haque et al. [16] found that the silane and compatibilizing agent treatments on fibre the surface showed initial improvement in interfacial properties, but, revealed a decrease in properties to the same value as the control fibres after 3 days of immersion within deionised water. CH_4_ plasma treatment of a CaP fibre/PGA composite studied by Ibnabddjalil et al. [17] revealed a 30% increase in interfacial shear strength; however, this decreased to even lower than the control fibres after immersion in phosphate buffered saline (PBS) for only a few hours. The treatments for PGF/polymer interface improvement as mentioned above rely on the chemical bond formed between fibre surface and the coupling agents or matrix. Additionally, as these composites are intended for implantation within the body, all coupling agents used must be biocompatible, which limits the selection.

Physical treatments can change the structural and surface properties of the fibre and thereby influence mechanical bonding with the matrix [18]. To date, no physical modification treatment has been investigated for the PGF/polymer composite system, whilst it has been used successfully in carbon fibre reinforced composites [6]. The objective of this study was to investigate improvement of the interfacial adhesion between PGF and polyester (polycaprolactone and polylactide) matrices through physical surface modification of the fibres. 

Magnetron sputtering is a promising coating technique and has been widely used to fabricate thin metal nanostructured films with varying morphologies and roughness [19]. For medical applications, magnetron sputtering has been used for coating CaP/HA onto medical devices to create a bioactive implant [20]. During the sputtering process, target atoms are sputtered by ions and neutrals, and a thin film layer is formed on the substrate surface. The process does not require any solvents and can be conducted at near room temperature. Magnesium alloys have been used as degradable implants in the clinic since 1878 and show good biocompatibility [21]. Previous studies on sputtered Mg films, which were conducted mainly for hydrogen storage applications, have shown that thin nanocrystalline Mg films were formed exhibiting a rough morphology [22]. The surface topography can easily be modified by adjusting the coating conditions [23, 24]. 

In this study, bioresorbable PGFs were coated with magnesium via magnetron sputtering, with the aim of modifying the PGF surface morphology to investigate improvement of the PGF/PLA or PGF/PCL interface. The crystalline textures and roughness profile of the coatings in this study were investigated using X-ray diffraction (XRD) and atomic force microscope (AFM), respectively, and coating morphologies were observed by scanning electron microscope (SEM). The effects of these coatings on the mechanical properties of PGFs were studied. The interfacial properties were investigated using single fibre fragmentation tests to examine the effects of physical modification using PGF/PLA or PCL single fibre composites.

## 2. Materials and Methodology

### 2.1. Phosphate Glass and Fibres Production

Sodium dihydrogen phosphate (Na_2_HPO_4_), calcium hydrogen phosphate (CaHPO_4_), phosphorous pentoxide (P_2_O_5_), magnesium hydrogen phosphate trihydrate (MgHPO_4_·3H_2_O), and iron phosphate dihydrate (FeHPO_4_·2H_2_O) precursors (Sigma-Aldrich, UK) were used without further purification. The precursors were weighed according to the composition 45% P_2_O_5_—16% CaO—24% MgO—11% Na_2_O—4% Fe_2_O_3_ (mol%), and the mixture was placed into a Pt-5% Au crucible type BC18 (Birmingham Metal Company, UK) and dried in at 350°C for 30 minutes, before being transferred to another furnace at 1100°C for 90 minutes. The molten glass was then poured onto a steel plate and left to cool to room temperature. 

Phosphate glass fibres (PGFs) (ca. 26 *μ*m in diameter) were manufactured via a melt-draw spinning process using an in-house built fibre drawing facility [25]. Pulling temperature and speed were adjusted to ca. 1250°C and ca. 1060 rpm. Both glass and glass fibres produced were kept in a desiccator before use.

### 2.2. Deposition of Magnesium Thin Films

Both glass slides (Fisher Scientific, UK) used for analysis and glass fibres were subjected to magnetron sputtering for the deposition of magnesium films. Samples were loaded onto a spindle shaped sample holder and placed into a magnetron sputtering rig with two circular superVac magnesium targets (76.2 mm, 99.99%, Testbourne Ltd, UK); one was placed above and one below the fibre samples. The sample holder rotated at a speed of 2 rpm. The nearest distance of the target to glass/fibre is 62 mm; the farthest distance is 128 cm. Magnesium thin films were deposited by sputtering these magnesium targets in an argon atmosphere using a DC magnetron system at 30 W. The system vacuum was 7 × 10^−6^ mbar, and the working pressure was 3.3 × 10^−3^ mbar. The target surface was cleaned prior to deposition by presputtering for 10 minutes at 30 W. Coating times of 10, 30, 60, and 120 minutes were investigated. The coating thickness was 0.8 *μ*m, 2 *μ*m, 4 *μ*m, and 9.5 *μ*m, respectively.

### 2.3. Characterisation of Magnesium Films

Film thicknesses were confirmed using a Philips JEOL XL30 scanning electron microscope (SEM) at an accelerating voltage of 20 kV. The roughness of the films deposited was obtained using Atomic Force Microscopy (AFM) in tapping mode. Magnesium distribution on the fibre surface was measured using Energy Dispersive X-ray (EDX) via mapping scans of the cross-sectional area of fibre. The crystal structure of the films was examined via X-ray diffraction performed on a D500 diffractometer using CuK*α* radiation (*λ* = 0.154 nm), operating at 40 kV and 25 mA with a step size of 0.02° and dwell time of 2 s from 20° to 90°. The texture coefficients of the Mg films as a function of coating time were calculated using the following formula:

(1) $$\begin{array}{c} T_{c}=\frac{I_{m}\left(hkl\right)/I_{0}\left(hkl\right)}{\left(1/n\right)\sum_{1}^{n}I_{m}\left(hkl\right)/I_{0}\left(hkl\right)}, \end{array}$$

where *I*_*m*_(*hkl*) is the measured relative intensity of the reflection from the (*hkl*) plane, *I*_0_(*hkl*) is the relative intensity from the same plane in a standard reference sample, and *n* is the total number of reflection peaks from coating. The grain size of the deposited Mg films was estimated from the following Scherrer formula:

(2) $$\begin{array}{c} D=\frac{0.9\lambda }{B\cos\theta }, \end{array}$$

where *B* is the corrected fullwidth at half maximum (FWHM) of a Bragg Peak, *λ* is the X-ray wavelength, and *θ* is the Bragg angle.

### 2.4. Characterisation of Coated Fibres

#### 2.4.1. Fibre Tensile Properties

Fibre tensile properties were measured using a sensitive linear tensile test facility (LEX810, Diastron Ltd, Japan) coupled with a laser diameter gauge (Mutitoyo Series 544 LSM-500S). The crosshead speed of the machine was 1 mm/min, and the load cell capacity was 2000 N. A minimum of 20 samples were tested, and the Weibull parameters of the fibre tensile properties were calculated using Minitab 16 (version 16.2.2).

#### 2.4.2. Interfacial Shear Strength (IFSS) Measurement

Two types of matrix, polylactide (PLA, 6201D NatureWorks Mw 90,000–120,000) and polycaprolactone (PCL, 181609 Sigma-Aldrich Mw ca. 65,000), were used in this study. The interfacial shear strength (IFSS) between fibre and (PCL or PLA) matrix was measured via the single fibre fragmentation test (SFFT). Single glass fibres were embedded between 80 × 20 mm films of polymer and then hot pressed (120°C for PCL matrix, 210°C for PLA matrix) at 10 bar for 10 minutes to make 0.25 mm thick single fibre composites. The single fibre composites were cut into 65 × 10 × 0.25 (*l* × *b* × *h*) mm dog-bone-shaped specimens. These dog-bone-shaped specimens were loaded axially in a tensile testing machine (Hounsfield series S testing machine, UK) using a 1 kN load cell and a crosshead speed of 1 mm min^−1^. After tensile testing, the specimens were observed under an optical microscope (Nikon Optiphot, Japan) in order to ascertain the number of fibre fragments generated. The Kelly-Tyson model [26] was then used to calculate the IFSS values:

(3) $$\begin{array}{c} \tau =\frac{\sigma_{f}d}{2l_{c}}, \end{array}$$

where *τ* is the IFSS value, *d* is the fibre diameter, and *σ*_*f*_ is the fibre strength at a length equal to the critical fibre length *l*_*c*_.

The critical fibre length (*l*_*c*_) was calculated by

(4) $$\begin{array}{c} l_{c}=\frac{4}{3}\bar{l},\\ \bar{l}=\frac{l_{0}}{N}, \end{array}$$

where $\bar{l}$ is the average fragment length, *l*_0_ is the gauge length, and *N* is the number of fibre fragments.

Fibre strength, *σ*_*f*_, can be calculated from the Weibull distribution as follows [27]:

(5) $$\begin{array}{c} \frac{\sigma_{0}}{\sigma_{f}}={\left(\frac{l_{c}}{l_{0}}\right)}^{1/m}, \end{array}$$

where *σ*_0_ is the fibre strength at a particular gauge length *l*_0_ and *m* is the Weibull modulus (in this study the gauge length investigated was 25 mm).

#### 2.4.3. Statistical Analysis

Statistical analysis was performed using the Prism software package (version 3.02, GraphPad Software, San Diego, CA, USA, http://www.graphpad.com/). A one-way analysis of variance was calculated with the Tukey multiple comparison posttest (*P* < 0.05) to compare the significance of change in one factor at one time point.

## 3. Results

### 3.1. Magnesium Film Characterisation

#### 3.1.1. Film Thickness and Roughness

**Figure 1:**
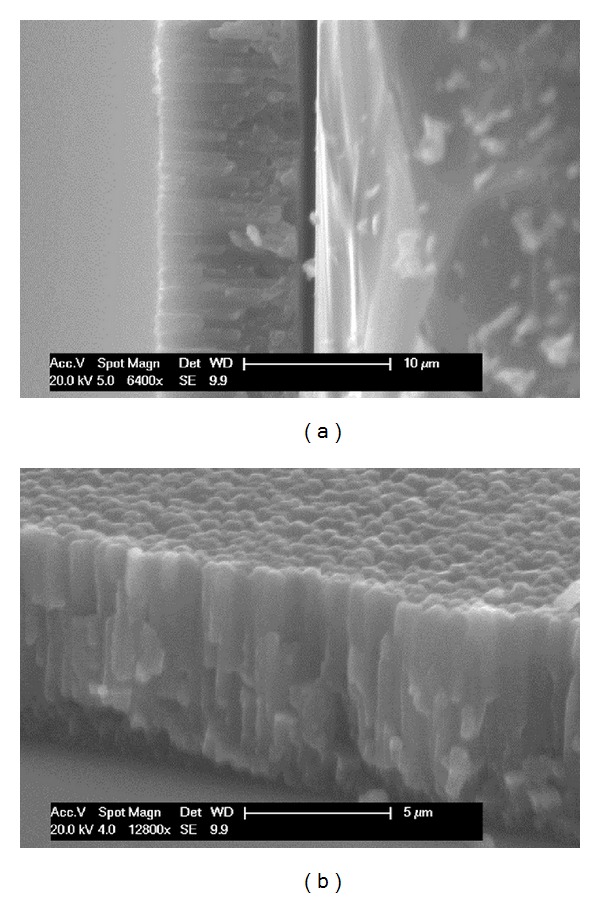
Transverse view of magnesium film deposition onto a glass slide after coating at 30 w for 120 min.

**Figure 2:**
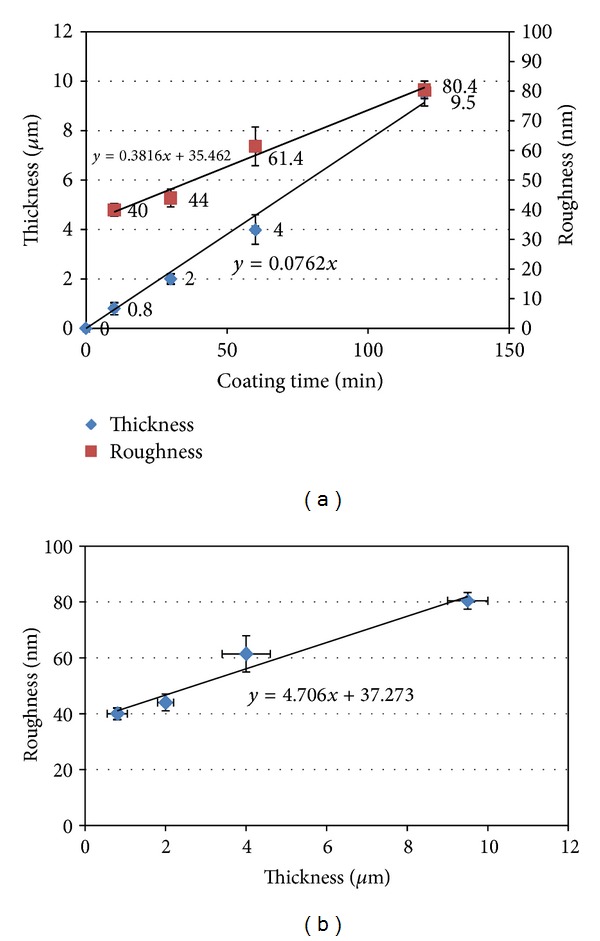
(a) Magnesium coating thickness and roughness variation with coating time, (b) the coating roughness variation with coating thickness.

**Figure 3:**
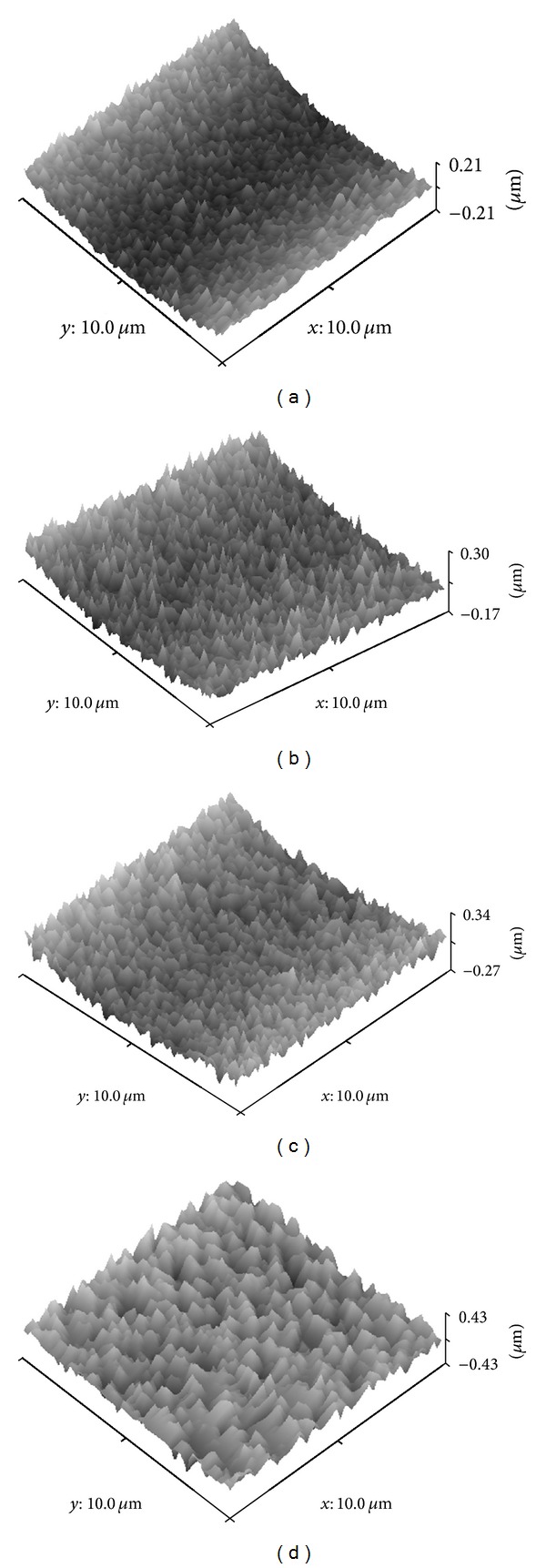
AFM of magnesium films deposited on glass slides at 30 W for (a) 10 min, (b) 30 min, (c) 60 min, and (d) 120 min.

Initially, thin magnesium films were deposited onto flat microscope glass slides in order to investigate their thickness and roughness features. The film thickness was measured from the cross-sectional view of the micrographs obtained. Representative transverse section images of the magnesium films deposited onto glass slides are presented in Figures 1(a) and 1(b) and revealed columnar growth structures. A linear increase in Mg film thickness from 0.8 *μ*m to 9.5 *μ*m with coating time (from 10 to 120 minutes) was shown in Figure 2. Film roughness measured by AFM was also observed to increase from 40 ± 1 nm to 80 ± 1 nm with increased coating time (3D topographical images obtained for magnesium films are shown in Figure 3). 

#### 3.1.2. Crystalline Structure

**Figure 4:**
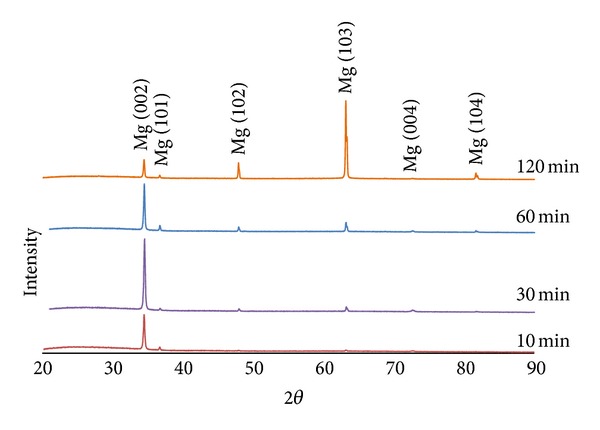
XRD of magnesium films deposited at 30 W.

**Figure 5:**
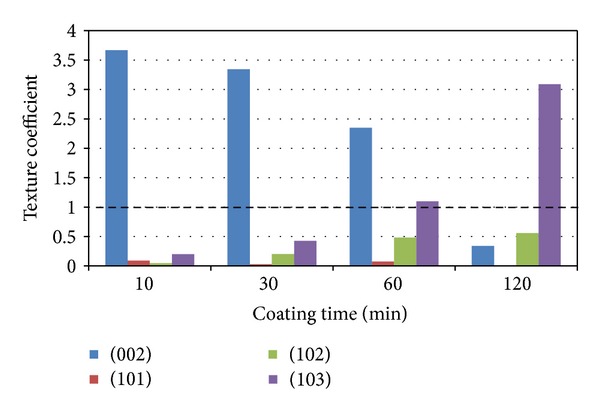
Texture coefficients of magnesium films deposited at 30 W. The dotted line represents the value of a randomly oriented sample.

XRD profiles for magnesium films deposited at 30 W are shown in Figure 4. A crystalline magnesium phase was identified for all the samples investigated (ICDD patent PDF-2 database File number 00-035-0821) with peaks observed at 2-theta values of 34.40, 47.83, 63.06, and 81.50. Figure 4 exhibited a (002) preferred orientation during the initial coating which then changed to a mixed orientation with more (102) orientation after 120 min coating. From the calculated texture coefficients, Figure 5, it was seen that the (002) orientation decreased with coating time, whereas the (102) and (103) orientations were seen to increase with coating time. 

### 3.2. Characterisation of Magnesium Film Deposition on Phosphate Glass Fibres

#### 3.2.1. Fibre Surface Morphology and Element Analysis

**Figure 6:**
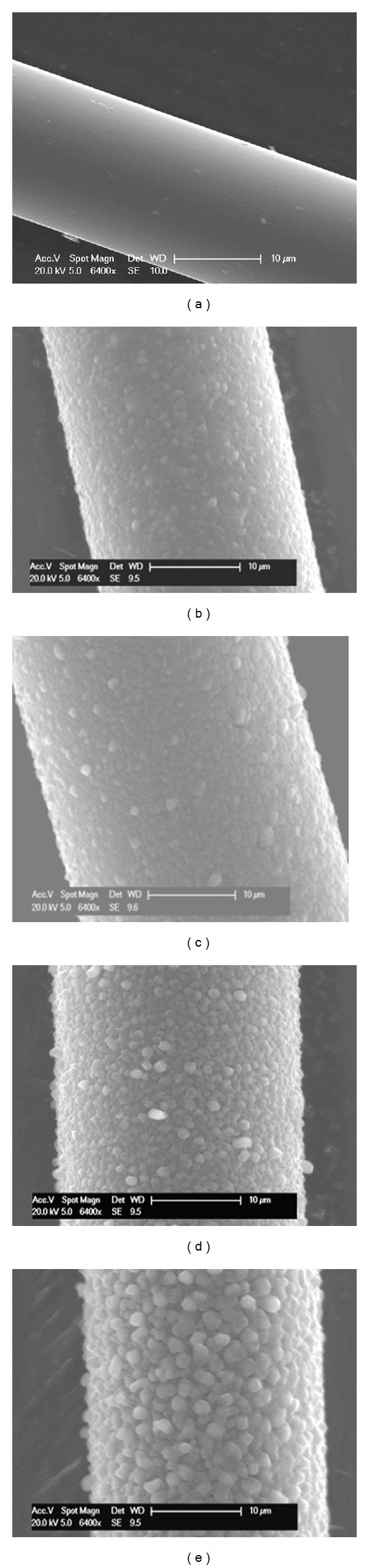
SEM of magnesium coated fibres with (a) pristine fibre, (b) 10 min, (c) 30 min, (d) 60 min, and (e) 120 min coating.

Phosphate glass fibres (PGF) were sputter coated with pure magnesium for 10, 30, 60, and 120 min. Sample surface morphologies observed under SEM are presented in Figures 6(a) to 6(e). The micrographs of the coated PGFs revealed a roughened surface morphology using a plasma sputtering process. Magnesium films deposited over time on the fibre surface showed separated granular morphologies in comparison with the continuous coatings observed on the glass slides (see Figure 1). It was seen that rougher films (compared with pristine fibre in Figure 6(a)) were created on the fibre surface with increased coating time which correlated well with the observations made for the coated glass slides (Section 3.1.1).

**Figure 7:**
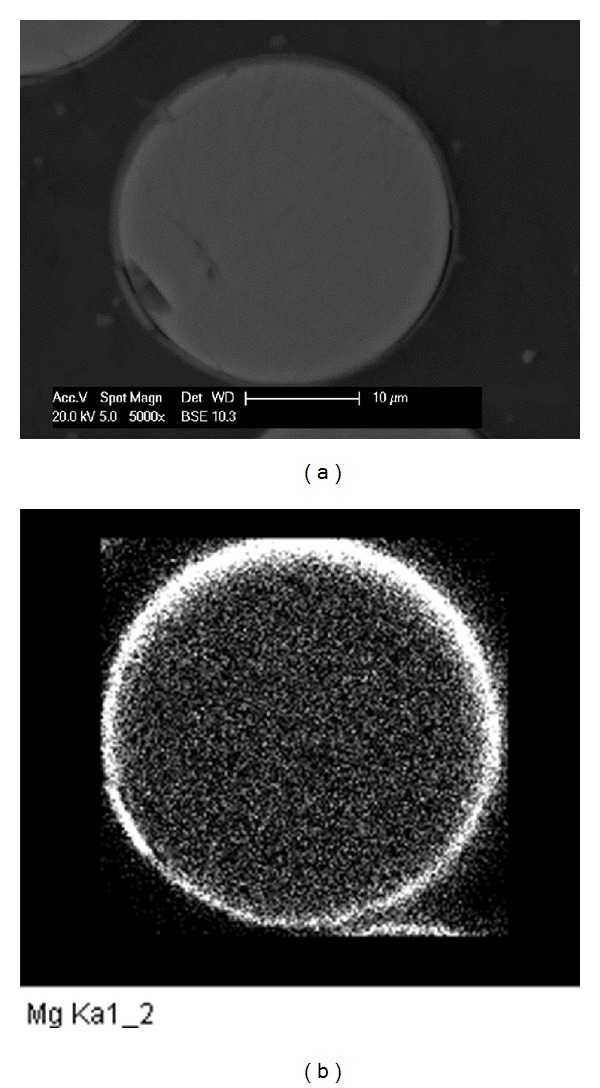
(a) SEM cross-sectional view of Mg coated fibre, (b) magnesium distribution observed using EDX in mapping mode.

Magnesium coated fibres were then embedded in casting resin and polished to observe their cross-sectional view. As seen in Figure 7(a), the fibres had an outer Mg coating layer that was clearly visible using backscattered SEM imaging. EDX analysis in mapping mode gave a clearer view of the magnesium distribution on the fibre cross-sectional area (see Figure 7(b)). A continuous magnesium coating was observed on the fibre periphery which was easily distinguished from the Mg contained within the glass formulation (seen as speckles within the white outer ring). The fibres were held at a radius of 33 mm from a rotating spindle such that the side of the fibre that faced the target was closer to the two targets, while the other side was 66 mm further away resulting in the observed variation of thickness. 

#### 3.2.2. Fibre Mechanical Properties

**Figure 8:**
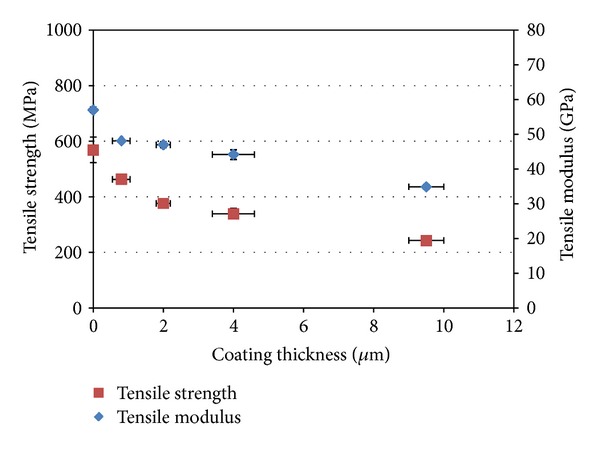
Mechanical properties of control fibre and coated fibres measured via the single fibre tensile test.

PGFs before and after coating were measured by single fibre tensile test. Tensile strength obtained decreased from 569 ± 46 MPa to 463 ± 15 MPa, whilst, the modulus decreased from 57 ± 1 GPa to 48 ± 0.2 GPa after 10 min coating (Mg coating thickness 0.8 *μ*m). Further reduction of properties was observed with increased coating time (see Figure 8). Fibre tensile strength decreased by 57% ± 6% after 120 min coating, whilst tensile modulus decreased by 38% ± 2%.

#### 3.2.3. Interfacial Shear Strength Test

The single fibre fragmentation test (SFFT) was used to investigate the interfacial properties between fibres and matrix. The constant shear model of Kelly and Tyson was used to calculate IFSS [26]. The validity of the Kelly and Tyson model for the calculation of IFSS from the fragmentation (SFFT) test requires that the samples are fully saturated with fragments [28]. Thus, before calculating IFSS with SFFT data, the numbers of fragments obtained were checked for full saturation using as received phosphate glass fibres.

**Figure 9:**
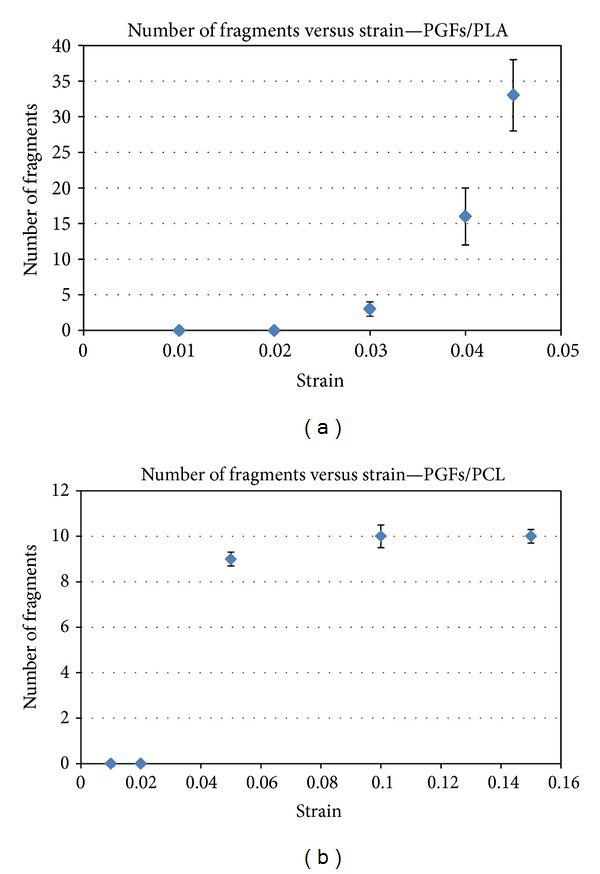
Number of fragments versus strain for (a) PGF/PLA and (b) PGF/PCL single fibre composites.

In this study, PLA and PCL were chosen as the matrix for the IFSS test in order to investigate if full saturation with fibre fragments could be achieved. The numbers of fragments obtained versus strain are shown in Figures 9(a) and 9(b). For the PLA single fibre composite samples, the number of fragments was seen to increase with increasing strain (Figure 9(a)). Increasing the strain further past 4.5% resulted in failure of the PLA matrix. However, the number of fragments increased with increasing strain and reached a plateau after 10% for PCL based samples (see Figure 9(b)). This observation confirmed that full saturation of fragments was achieved using PCL, thus PLA was not used for further investigations.

**Figure 10:**
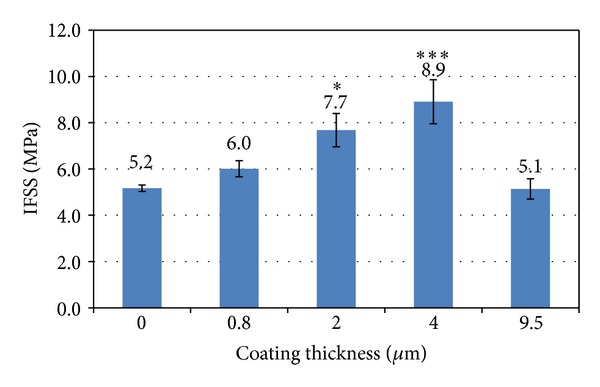
Comparison of IFSS value of control fibre and coated fibres measured by single fibre fragmentation test (the star symbols show significance value: one star (*) for *P* < 0.05 and three (***) for *P* < 0.001).

As fibre strength was seen to decrease after magnesium coating (Figure 8), the Weibull parameters (shape and scale) used to calculate IFSS were obtained using their real tensile strength data (i.e., from the coated fibre properties at each coating time). The Weibull scale values for magnesium coated (10, 30, 60, and 120 min) fibres were 490.1, 405.3, 371.8, and 264.5, respectively. Whilst the Weibull shape values obtained were 8.3, 5.6, 4.6, and 4.6, respectively. Utilising these Weibull parameters, the IFSS values calculated for the magnesium coated fibres are shown in Figure 10. The highest value achieved was approximately 8.9 ± 1 MPa obtained for the 4 *μ*m Mg coated samples. From one-way ANOVA analysis, significant difference was observed from 2 *μ*m (*P* < 0.05) and 4 *μ*m (*P* < 0.001) coating as compared with the control single fibre composite. 

## 4. Discussion 

In this study, bioresorbable PGFs were coated with degradable magnesium via magnetron sputtering to try and create a rougher fibre surface in order to initiate a mechanical interlock between the fibre and matrix, thus improving the composite interfacial properties. The roughness and thickness of these deposited magnesium films were seen to increase linearly with increasing coating time as shown in Figure 2(a).  Figure 2(b) shows that the roughness varies with thickness over this range of 40–80 nm. This is typical of sputtered coatings [23]. The coatings columnar structure, Figure 1(b), is typical of magnesium coatings by PVD [24]. 

However, applying the same coating conditions as above to the surface of the PGFs revealed a different morphology. The magnesium coating generated on the outer layer of the PGFs showed a roughened surface with separated granular morphology. It is suggested that the morphology difference observed may have been due to the effect of the incidence angle of the sputtered Mg from the target. Due to the curved shape of the fibre and the rotation of the fibre on the spindle, the incidence angle varies at any one spot on the fibre with time unlike a flat substrate in which the incidence angle would be constant. It has been shown that the angle of incidence of a substrate to a sputtered magnesium target strongly influences the preferred crystal growth direction. Störmer et al. [29] studied Mg coating on a silicon wafer with varied deposition angle from 0° to 70°. It was found that the coating morphology was strongly depends on the deposition angle and argon pressure. Increased roughness of Mg coating with higher deposition angle was observed. In the experiment presented here the angle was constantly changing thus the preferred orientation was also changing resulting in less orientation specific coating, with a more granular appearance from above and disrupted boundaries between grains.

XRD analysis of the Mg films on the glass slides revealed a hexagonal closed packed (hcp) structure. A highly intense (002) orientation (at 34.40 2-theta) peak was observed, which was suggested to be due to the low surface energy configuration corresponding to the (002) plane [23]. Competition between strain energy and surface energy during film growth may also have contributed to changes in preferred orientation during deposition [30]. The thickness increase of the coating led to additional strain energy resulting in the (102) and (103) orientations dominating to reduce the total energy in the system. The difference in growth rates for the different crystal planes created a roughened surface texture [31]. 

**Figure 11:**
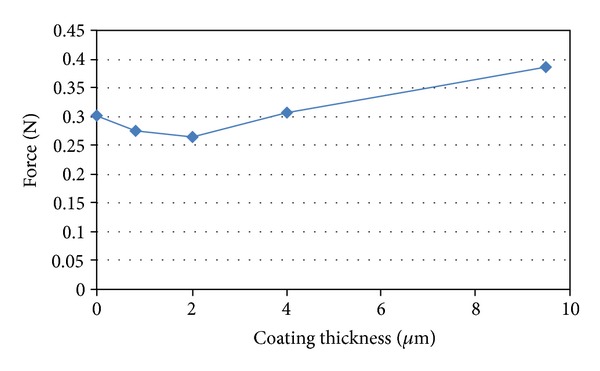
The breaking force for Mg coated fibres versus coating thickness.

Mechanical testing of the fibres with coating (via single fibre tensile tests) showed a significant reduction (*P* < 0.05) in strength and modulus after coating with magnesium. The breaking force for Mg coated fibres versus coating thickness seen in Figure 11 showed a decrease of force during initial coating which indicated that the fibres must have been damaged during the early stages of the coating process. According to Griffith's theory of brittle fracture [32], the strength of glass fibres is related to the fine cracks or flaws on the fibre surface. When fibres are under tension, stress concentrates at these fine cracks, and the cracks propagate into brittle fractures. During the coating process, magnesium atoms were sputtered towards the fibre surface at several hundred eV. It is suggested that this type of atomic bombardment may have created flaws on the fibre surface or potentially enlarged the already existing inherent flaws. Moreover, the thermal expansion coefficient for a phosphate glass with a formulation similar to the one used in this study has been reported as 12 × 10^−6^ K^−1^ [33], whilst for pure magnesium a thermal expansion coefficient of 28.4 × 10^−6^ K^−1^ [34] was reported. As such, this difference in thermal expansion coefficients may have contributed additional stress concentrations during the coating process, as heat produced within the chamber during the sputtering process may have resulted in contraction of the Mg coating more so than the fibre upon cooling. This could have placed the fibre surface under compression and the internal fibre under tension leading to the fibre being more susceptible to cracking and ultimately resulting in a decrease of the properties as observed [35]. 

It was suggested that flaws were created on the fibre surface during the early stages of coating which resulted in the mechanical property decrease. Once the coating had reached a certain thickness, the decreasing mechanical properties were due to Mg deposition, as Mg has lower mechanical properties than phosphate glass fibre. Bulk Mg has a modulus of 45 GPa. However, the sputtered Mg coating in this study contained voids, thus the mechanical properties were expected to be even lower.

Characterisation of the interfacial properties between the fibre and matrix was conducted using the single fibre fragmentation test (SFFT) and Kelly Tyson model [26]. In order to use the Kelly Tyson model, the fibre fragments of the samples should be fully saturated before failure of the matrix. In addition, as a general guideline, in this model the strain required for saturation should be at least three times the fibre failure strain. However, a study by Netravali et al. [36] observed that the strain required for saturation depended on the ductility of the embedding resin. Thus, in this study, two bioresorbable polymer matrices (PLA and PCL) were chosen as matrix and investigated. Single fibre composites of PGF/PLA and PGF/PCL were prepared and tensile tested at varying strains to investigate saturation effects. The maximum critical strain obtained using PLA was approximately 4.5% due to its intrinsic brittle nature. Further strain resulted in matrix failure before saturation with fibre fragments had occurred. However, using PCL strain limits of approximately 15% was obtained, due to the ductile properties of PCL. As seen from Figure 9, a plateau in fibre fragments was seen at 10% strain for PCL. This result revealed that the SFFT was not suitable for the PGF/PLA system (using NatureWorks 6201D PLA) in order to determine the IFSS value. Thus, only the SFFT using the PGF/PCL system was deemed suitable from which to calculate IFSS. 

From the interfacial studies conducted, an increase in IFSS properties was seen with an increase in 4 *μ*m thick Mg coated PGF using the PGF/PCL system. Significant increase was seen in 2 *μ*m and 4 *μ*m coating, which resulted in a 48% and 72% higher IFSS value, respectively. This increase in IFSS was suggested to be due to the rough fibre surface creating a stronger interlock with the polymer matrix. Studies by Lin et al. [6] showed a 110% increase in interfacial strength for ZnO nanowire coated carbon fibre which was suggested to be due to the ZnO nanowires penetrating the epoxy matrix creating a strong interlock, and further modification of the nanowire dimensions resulted in the interfacial strength increasing by up to 228% [37]. In addition, an increase in surface area (as evidenced by increasing surface roughness) at the fibre surface would also have contributed to enhancing the fibre and matrix contact. The IFSS value was seen to decrease to a similar value as the control for the (longer) 120 min coating time (9.5 *μ*m coating) which indicated that another mechanism dominates, such as the energy of bombardment due to the Mg deposition and/or the elevated temperature in the chamber deteriorating the fibre properties.

The work presented here showed that a roughened fibre surface increased the IFSS properties of a single fibre composite; however, the effect of coating parameters on the fibre surface (especially regarding the type of bonding between the coating and fibre surface) requires further investigation. In addition, prevention of possible damage to the fibres during the coating process also needs to be explored.

## 5. Conclusions 

Magnesium thin films were deposited onto glass slides and bioresorbable phosphate glass fibres using magnetron sputtering. Four different coating times were investigated, and both roughness and thickness were seen to increase with increased coating time. Mg coating thicknesses of 0.8–9.5 *μ*m were deposited on the phosphate glass fibre surface. A columnar structure was observed for the magnesium coating, and crystalline phases of magnesium were detected, with growth preferred in the (002) orientation in 0.8 *μ*m coating gradually changed to mixed orientations in (002), (102), and (103) planes with 9.5 *μ*m coating. The coated fibre mechanical properties (tensile strength and modulus) were seen to decrease with increased magnesium coating time. The decrease in the early stages of coating was suggested to be due to damage to fibre surface caused by Mg particle bombardment, and further reduction with Mg deposition was caused by the lower property of Mg coating. The IFSS values of PGFs/PCL single fibre composites increased after magnesium coating. A statistically significant increase in IFSS was seen with 2 and 4 *μ*m magnesium coating (*P* < 0.05). Whilst, it was found that PGF/PLA (NatureWorks 6201D) single fibre composites had insufficient strain for single fibre fragmentation testing. Future investigations will focus on the prevention of damage to the fibre mechanical properties during the coating process.
